# Supplementary figures and images for: Metabolome and Transcriptome Integrated Analysis of Mulberry Leaves for Insight into the Formation of Bitter Taste
Source: Genes (Basel). 2023 Jun 17;14(6):1282. doi: 10.3390/genes14061282 (PMC10298079; doi:10.3390/genes14061282)

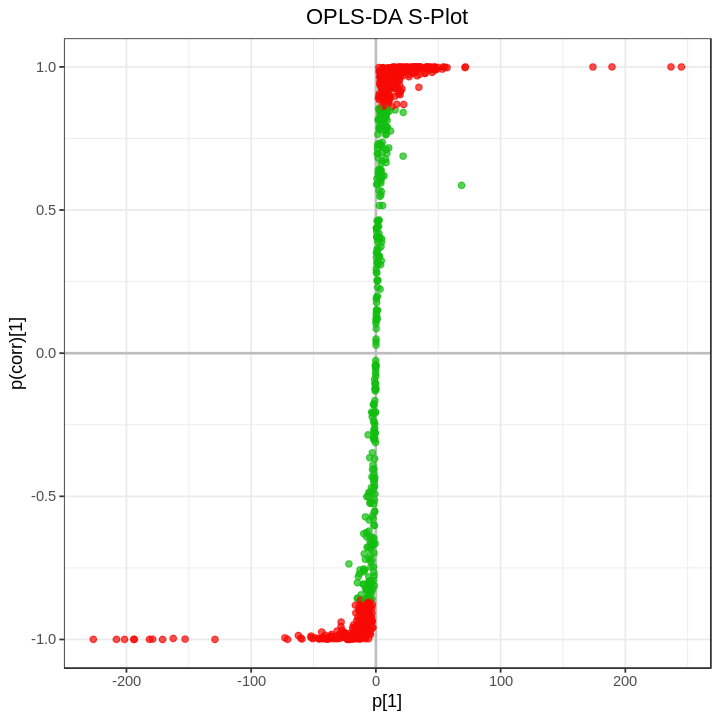

Supplement: Supplementary file 1 [file genes-14-01282-s001.zip › Figure S1.png]

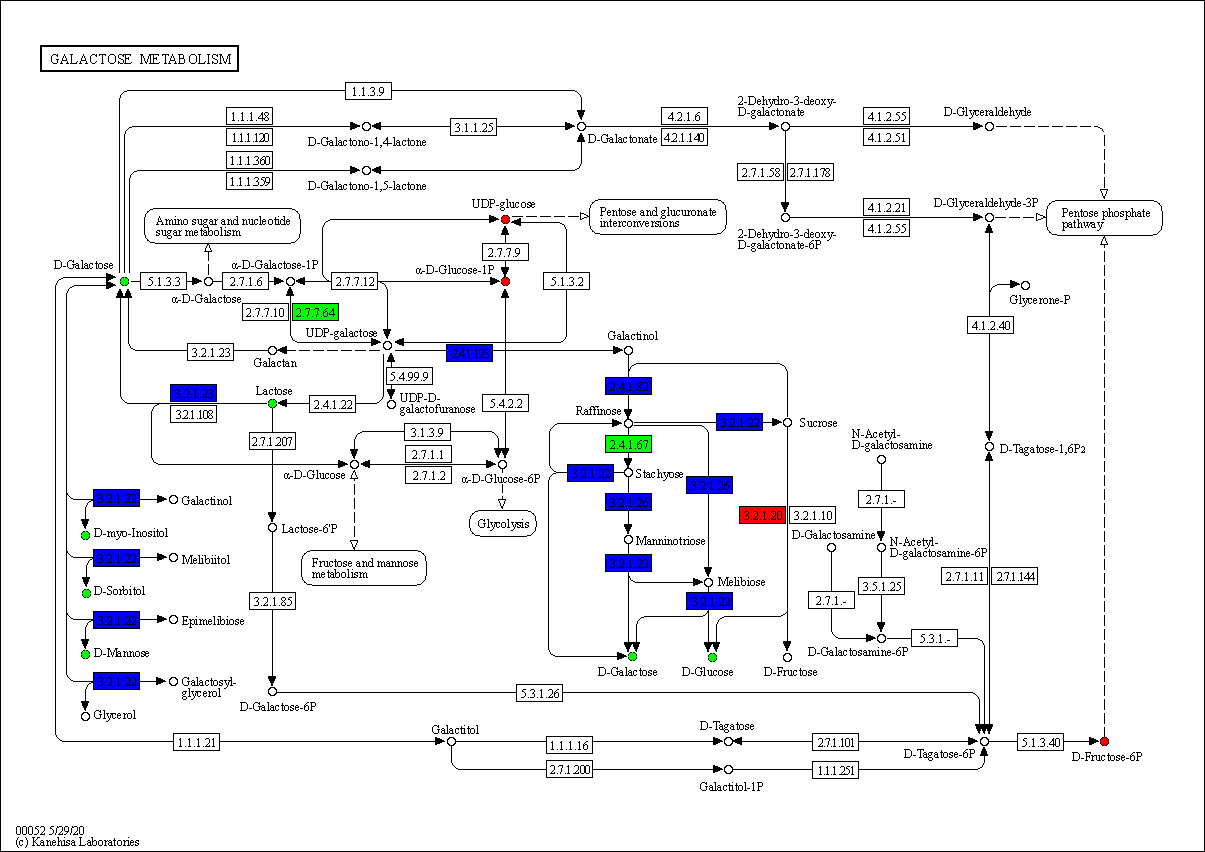

Supplement: Supplementary file 1 [file genes-14-01282-s001.zip › Figure S2.png]
